# What is normal? Apelin and VEGFA, drivers of tumor vessel abnormality

**DOI:** 10.15252/emmm.201910892

**Published:** 2019-07-18

**Authors:** Lena Claesson‐Welsh

**Affiliations:** ^1^ Science for Life and Beijer Laboratories Department of Immunology, Genetics and Pathology Uppsala University Uppsala Sweden

**Keywords:** Cancer, Vascular Biology & Angiogenesis

## Abstract

In this issue of *EMBO Molecular Medicine*, Uribesalgo and coworkers show that high Apelin expression correlates with poor survival in advanced breast (MMTV‐*NeuT*) and lung (*KRAS* ^*G12D*^) murine tumor models as well as in breast and lung cancer in humans. Combining Apelin inhibition (genetically or using an inactive Apelin agonist) with anti‐angiogenic therapy using different small molecular weight kinase inhibitors (sunitinib, axitinib) led to marked delay in breast cancer growth in mice. The vasculature in Apelin‐targeted cancer showed normalized features including improved perfusion and reduced leakage. These important data provide a strong incentive to target Apelin in human cancer treatment.

Apelin consists of 13‐ to 36‐residue peptides that bind to the G protein‐coupled receptor APJ (Lee *et al*, 2000). Apelin belongs to the adipokine family released by adipose tissue, but is expressed by many different cell types. APJ is preferentially expressed by cardiomyocytes and endothelial and vascular smooth muscle cells (Chen *et al*, 2003). Mice with a constitutive deletion of the *Apln* gene are viable and fertile. Still, Apelin/APJ have been implicated in many physiological processes including cardiac function, body fluid homeostasis, angiogenesis, and energy metabolism with bearing for pathological conditions such as heart failure, obesity, diabetes, and cancer (for a review see Wysocka *et al*, 2018).

While Apelin by itself has a minimal effect on angiogenesis during normal development such as the retina vasculature, it causes excessive growth of dilated and tortuous vessels in retinopathies (McKenzie *et al*, 2012). However, in apparent contrast, Apelin enhances lymphatic and blood vessel integrity in ischemic conditions, and in obesity, it blocks an increase in permeability induced by dietary fatty acids (Chen *et al*, 2003). The exact role of Apelin in regulation of endothelial function has therefore remained unclear.

Cancer is associated with a dysfunctional, morphologically abnormal vasculature characterized by poor vessel stability, obliterated lumen, and excessive leakiness. The broken vascular barrier facilitates tumor cell extravasation and seeding of distant metastasis. The underlying cause of the aberrant tumor vasculature is commonly the production of the hypoxia‐regulated vascular endothelial growth factor (VEGF), in particular VEGFA. Neutralizing antibodies targeting VEGFA or the ligand binding site on its receptor, VEGFR2, have been available in the clinic for more than a decade by now and have been tested in many different cancers with relatively limited clinical benefit overall (Martin *et al*, 2019). Small molecular weight tyrosine kinase inhibitors have been more successful perhaps since they as a rule act on several related receptor tyrosine kinases (for a review, see Yunus *et al*, 2019). New strategies to optimize anti‐angiogenic therapy are urgently needed.

The concept of vessel normalization in cancer therapy, first described by Rakesh Jain and colleagues in 2001, rests on the notion that tumor vessels have an abnormal morphology and function, compared to vessels in healthy tissues. Common abnormal features of tumor vessels include high vessel density, compressed and obliterated lumen, and increased leakiness, which all contribute to poor circulation and hypoxia. While it is unfavorable to completely suppress the tumor vasculature through vigorous anti‐angiogenic therapy as it further elevates tumor hypoxia, it may seem counter‐intuitive to therapeutically strive for tumor vessel normalization, as it would lead to better tumor oxygenation and possibly enhanced tumor growth. On the other hand, to relieve the tumor of hypoxia is likely to decrease its invasive behavior. The improved vessel barrier would further diminish tumor dissemination and also reduce the interstitial pressure, allowing more efficient penetrance of drugs.

To exploit tumor vessel normalization clinically, we need to better understand the components of vessel abnormality. Are there differences between vessels in different tumor types, in different organs, with regard to the underlying molecular mechanisms leading to the abnormal morphology and function of tumor vessels? Many cell types in the tumor microenvironment including tumor cells themselves, cancer‐associated fibroblasts, perivascular cells, and immune cells may contribute to the abnormality, for example, by secretion of cytokines including but not limited to VEGFA (Martin *et al*, 2019). A key question is therefore how stable normalization can be achieved and whether cancer‐specific strategies for stable normalization must be employed. To suppress VEGFA/VEGFR2 is clearly not sufficient and may even lead to excessive pruning, increased hypoxia, and worsened disease progression.

The clinical relevance of the report by Uribesalgo *et al* is underscored by that high *Apln* transcript levels in breast cancer correlates with metastatic spread. In renal cell carcinoma, combined low levels of VEGF and Apelin in sunitinib‐treated patient serum correlated with a 6‐fold longer progression‐free survival compared to patients with high levels of both VEGF and Apelin. The effect of suppressing Apelin expression in cancer may however be dependent on the cancer type. In glioma, Apelin depletion resulted in increased tumor invasive behavior (Mastrella *et al*, 2019). However, in agreement with the results from the Uribesalgo study, suppression of Apelin function combined with anti‐angiogenic therapy resulted in efficient decrease in glioma progression (Mastrella *et al*, 2019). On the other hand, studying the effect of overexpression of Apelin in murine colon26 adenocarcinoma and Lewis lung carcinoma, Kidoya *et al* showed impaired tumor growth and reduced tumor vessel permeability as a consequence of Apelin overexpression (Kidoya *et al*, 2012). These data stress that it is critical to test mediators of vessel normalization for cancer‐type‐specific effects. Also, we need to learn more about how Apelin suppression is favorable in a VEGFR2‐inhibited setting as Apelin at least in part seems to act by suppressing the VEGFA transcriptome (Uribesalgo *et al*, 2019), which to a major extent should be VEGFR2‐dependent. Finally, we need to understand whether suppressing Apelin is beneficial when combined with checkpoint inhibitor‐based immunotherapy.

The important data from Uribesalgo *et al*, summarized in Fig 1, provide a foundation for the development of guidelines for personalized treatment of cancer such as in the breast and lung where anti‐angiogenic therapy should be used preferentially on patients carrying tumors expressing low levels of Apelin. The clinical implementation is facilitated by the fact that circulating Apelin levels serve as a biomarker for the cancer's sensitivity to anti‐angiogenic treatment. In the anticipation of novel Apelin‐targeting drugs, patients with high levels of Apelin in their tumors on the other hand do not seem to benefit from the expensive and the challenging anti‐angiogenic treatment. For these patients, yet other novel therapies are needed.

**Figure 1:**
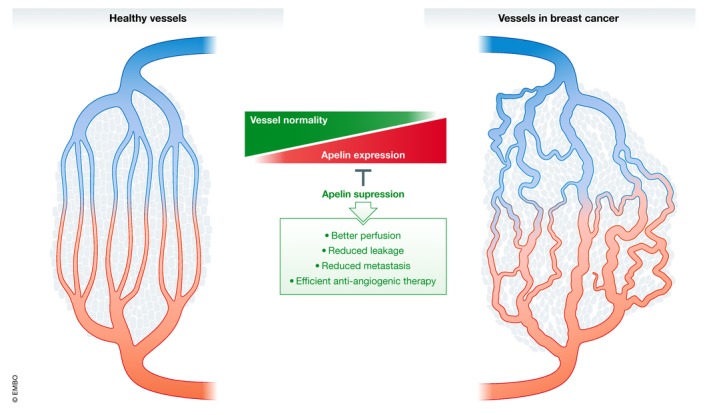
Unlike vessels in the healthy tissue, tumor vessels are abnormal, that is, tortuous, obliterated, and leaky, contributing to tumor inflammation, tumor invasion, and metastatic spread Expression of Apelin correlates with tumor vessel abnormality, and suppression of Apelin improves vessel perfusion and reduces leakage and metastatic spread
